# Dynamic Electrophoresis of an Oil Drop

**DOI:** 10.3390/mi16121407

**Published:** 2025-12-15

**Authors:** Hiroyuki Ohshima

**Affiliations:** Research Institute for Science and Technology, Tokyo University of Science, 2641 Yamazaki, Noda, Chiba 278-8510, Japan; ohshima@rs.noda.tus.ac.jp

**Keywords:** dynamic electrophoresis, dynamic electrophoretic mobility, oil drop, Marangoni effect

## Abstract

We present a theoretical framework describing how the electrophoretic mobility of a weakly charged oil droplet in an aqueous electrolyte varies with frequency when the system is subjected to an oscillatory electric field. The surface charge of the droplet arises from the adsorption of electrolyte ions. Our analysis is based on a simplified form of the Baygents–Saville model, in which the interior of the droplet is assumed to contain no dissolved ions. In this approach, variations in interfacial tensions along the droplet surface, generated by the Marangoni effect, are explicitly included. From the formulation, we derive a general expression for the dynamic electrophoretic mobility of a charged spherical droplet, and, in addition, obtain concise analytical formulas applicable in the limit of small zeta potentials.

## 1. Introduction

According to the DLVO theory for the stability of colloidal dispersions [1,2,3], the zeta potential of particles, including liquid drops, plays an essential role [4,5,6,7,8,9,10,11,12,13,14,15,16,17,18,19,20,21,22]. The zeta potential is usually determined from the electrophoretic mobility, which can be measured by either static methods such as conventional electrophoresis or dynamic methods such as electroacoustic measurements.

Electroacoustic techniques such as the colloid vibration potential (CVP) and the electrokinetic sonic amplitude (ESA) have long been used to probe colloidal dispersions [23,24,25,26,27,28,29,30,31]. These phenomena provide indirect yet valuable access to key interfacial properties of colloidal entities, particularly the particle zeta potential, even in concentrated or opaque systems where optical approaches such as light scattering are not feasible. In an alternating electric field, oscillatory particle motion generates acoustic waves (ESA), while conversely, a propagating sound wave induces an electric signal (CVP). Both effects are inherently linked through the dynamic electrophoretic mobility of the suspended particles.

Compared with rigid particles, soft or fluid inclusions such as emulsified oil drops, gas bubbles, or biological cells present additional complexities [32,33,34,35,36,37,38,39,40,41,42,43,44,45,46,47,48,49,50,51,52]. The flow fields both inside and outside the drop must be solved simultaneously, with interfacial conditions that account for Marangoni stresses arising from surface tension gradients [35].

These distinctive features suggest that electroacoustic effects in suspensions of dielectric drops may differ fundamentally from those in rigid or conducting systems.

Recent studies have also explored a wide range of alternating-current electrokinetic phenomena, including induced-charge electrokinetic self-propulsion of metallodielectric Janus particles and nonlinear effects in insulator-based dielectrophoresis [53,54]. These investigations highlight the rich behavior that can arise in AC electric fields, especially when induced charges or Joule-heating-driven nonlinearities are present. Although the present work focuses on the linear dynamic electrophoresis of weakly charged, spherical oil drops, it may provide a useful reference framework for understanding AC electrokinetic responses of soft particles in general.

In our recent series of studies [55,56,57,58], we constructed a theoretical description of the static electrophoretic mobility of weakly charged oil drops dispersed in electrolyte solutions, drawing on the Baygents–Saville framework [35], which models interfacial charging through ion adsorption and includes Marangoni stresses. In that formulation, the interfacial charge was attributed to the adsorption of ions, and a simplified version of the Baygents–Saville model was used, where ions inside the drop were neglected while the influence of surface-tension gradients was retained. These studies yielded analytical expressions for the static mobility of ion-adsorbed drops [55] and further demonstrated that the Onsager reciprocal relation connects electrophoresis and the sedimentation potential in suspensions of such liquid drops [56].

In the present work, we build upon that earlier theory [55,56,57,58] and generalize it from the static case to the dynamic case, focusing on the frequency-dependent electrophoresis of oil drops within the same simplified Baygents–Saville framework [35]. We obtain a general expression for the dynamic mobility of a charged spherical drop and further derive a compact analytical formula applicable to weakly charged oil drops in aqueous electrolyte environments.

## 2. Theory

### 2.1. Governing Equations

We examine the motion of a spherical oil drop bearing an electric charge, with radius a, mass density $\rho_{p}$, viscosity $\eta_{d}$, and zeta potential ζ. The drop oscillates with a velocity $Ue^{-i\omega t}$ in an aqueous medium characterized by relative permittivity $\varepsilon_{r}$, mass density $\rho_{0}$, and viscosity η. The surrounding solution contains a general electrolyte composed of N ionic species, each with valence $z_{j}$, bulk number density $n_{j}^{\infty }$, and friction coefficient $\lambda_{j}\left(j=1,2,\ldots ,N\right)$. The system is subjected to an external electric field of the form $Ee^{-i\omega t}$, where ω is the angular frequency, i is the imaginary unit, and t denotes time.

**Figure 1:**
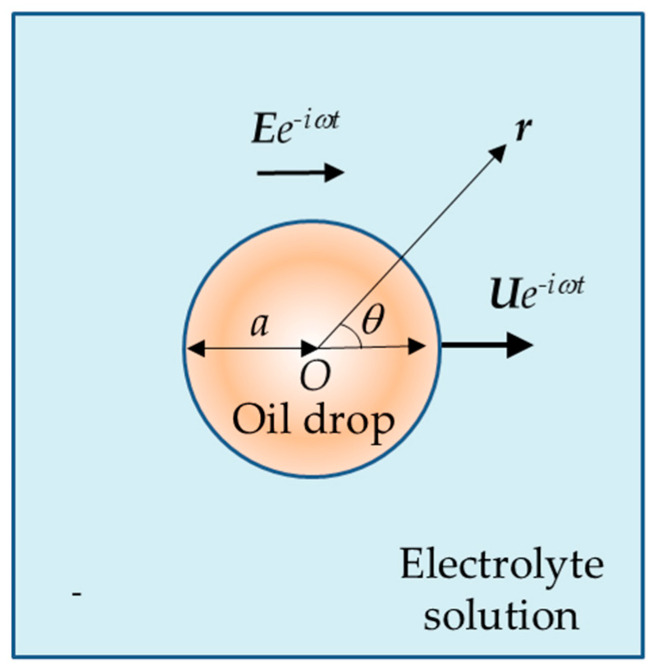
Schematic illustration of a spherical oil drop of radius *a* suspended in an aqueous electrolyte solution under an oscillating electric field ***E**e^−iwt^*. The electric field vector is shown pointing to the right, representing an instantaneous direction of the applied AC field. The drop velocity ***U**e^−iwt^* is also indicated in the same direction for simplicity, although in reality the drop motion may lag in phase behind the applied electric field. The position vector ***r*** and the polar angle *θ* are also shown.

A schematic representation of the situation is provided in Figure 1. The figure shows a charged oil drop of radius a dispersed in an electrolyte solution and exposed to the time-dependent electric field $Ee^{-i\omega t}$. The electric field vector is illustrated pointing to the right to indicate the instantaneous direction of the imposed field. For clarity, the drop velocity $Ue^{-i\omega t}$ is drawn along the same axis, although, in practice, the drop’s response generally lags behind the applied electric field in phase.

The frequency-dependent dynamic electrophoretic mobility *μ*(*ω*) of the drop is defined by

(1) $$U=\mu \left(\omega \right)E$$

We adopt the theoretical framework of Baygents and Saville [35], which describes drops with a surface charge originating from ion adsorption and includes the Marangoni effect due to the surface tension gradient.

The present analysis is based on the following key assumptions. (i) The Reynolds numbers for fluid motion both inside and outside the droplet are sufficiently small that inertial terms in the Navier–Stokes equations can be neglected, and the fluid may be treated as incompressible. (ii) The applied electric field ***E*** is weak enough that the drop velocity ***U*** is directly proportional to ***E***, and higher-order effects in ***E*** can be disregarded. (iii) The drop remains spherical when subjected to the electric field ***E***, which is justified if the interfacial tension is sufficiently large. (iv) Electrolyte ions are excluded from the drop interior. (v) Adsorption of ions onto the drop surface occurs in a linear fashion, with the surface concentration of adsorbed ions expressed as the product of the local bulk ion concentration and an adsorption constant.

The origin of the spherical polar coordinate system (*r*, *θ*, *φ*) is fixed at the center of the drop, which moves with velocity ***U***(*U*cos*θ*, −*U*sin*θ*, 0). The polar axis (*θ* = 0) is taken parallel to ***E***(*E*cos*θ*, *−E*sin*θ*, 0) and *U* and *E* represent the signed magnitude of ***U*** and the magnitude of ***E***, respectively. The electrolyte solution consists of *N* mobile ionic species, each having a valence *z_j_*, a bulk number concentration $n_{j}^{\infty }$, and a drag coefficient *λ_j_* (*j* = 1, 2, …, *N*). The electroneutrality condition of the system is given by $\sum_{j=1}^{N}z_{j}{en}_{j}^{\infty }=0$, which ensures that the total charge of all ionic species in the bulk electrolyte is zero.

The governing electrokinetic equations describe the fluid velocity ***u***(***r***, *t*) = (*u_r_*(***r***, *t*), *u_θ_*(***r***, *t*), 0) in the region outside the drop, the internal flow field ***u***_I_(***r***, *t*) = (*u*_I_*_r_*(***r***, *t*), *u*_I_*_θ_*(***r***, *t*), 0), inside the drop, and the velocity $v_{j}\left(r,t\right)={(v}_{jr}\left(r,t\right),v_{j\theta }\left(r,t\right),0)$ of the *j*-th ionic species (*j* = 1, 2, …, *N*) are given as follows. Because the system is axially symmetric with respect to the polar axis (*θ* = 0), all these variables depend only on *r*, *θ*, and *t*.

(2) $$u\left(r,\theta ,t\right)=u\left(r,\theta \right)e^{-i\omega t}$$

(3) $$u_{I}\left(r,\theta ,t\right)=u_{I}\left(r,\theta \right)e^{-i\omega t}$$

(4) $$v_{j}\left(r,\theta ,t\right)=v_{j}\left(r,\theta \right)e^{-i\omega t},(j=1,\;2,\;\cdots ,N)$$

The governing electrokinetic equations for these velocities are

(5) $$\rho_{0}\frac{\partial }{\partial t}\left\{u\left(r,\theta ,t\right)+Ue^{-i\omega t}\right\}+\eta \nabla \times \nabla \times u\left(r,\theta ,t\right)+\nabla p\left(r,\theta ,t\right)+\rho_{e}\left(r,\theta ,t\right)\nabla \psi \left(r,\theta ,t\right)=0$$

(6) $$\rho_{d}\frac{\partial }{\partial t}\left\{u_{I}\left(r,\theta ,t\right)+Ue^{-i\omega t}\right\}+\eta_{d}\nabla \times \nabla \times u_{I}\left(r,\theta ,t\right)+\nabla p_{I}\left(r,\theta ,t\right)=0$$

(7) $$\nabla \cdot u\left(r,\theta ,t\right)=0$$

(8) $$\nabla \cdot u_{I}\left(r,\theta ,t\right)=0$$

(9) $$v_{j}\left(r,\theta ,t\right)=u\left(r,\theta ,t\right)-\frac{1}{\lambda_{j}}\nabla \mu_{j}\left(r,\theta ,t\right)$$

(10) $$\mu_{j}\left(r,\theta ,t\right)=\mu_{j0}+z_{j}e\psi \left(r,\theta ,t\right)+kTln\left[n_{j}\left(r,\theta ,t\right)\right]$$

(11) $$\frac{\partial n_{j}\left(r,\theta ,t\right)}{\partial t}+\nabla \cdot \left\{n_{j}\left(r,\theta ,t\right)v_{j}\left(r,\theta ,t\right)\right\}=0$$

(12) $$∆\psi \left(r,\theta ,t\right)=-\frac{\rho_{e}\left(r,\theta ,t\right)}{\varepsilon_{r}\varepsilon_{0}}$$

(13) $$\rho_{e}\left(r,\theta ,t\right)=\sum_{j=1}^{N}z_{j}n_{j}\left(r,\theta ,t\right)$$

(14) $$∆\psi_{I}\left(r,\theta ,t\right)=0$$

Here *p*(*r*, *θ*, *t*) and *p*_I_(*r*, *θ*, *t*), respectively, denotes the pressure outside and inside the drop, *ρ*_e_(*r*, *θ*, *t*) represents the charge density arising from the mobile ionic species, as defined in Equation (13). The electric potential in the region outside the drop is expressed as *ψ*(*r*, *θ*, *t*). The symbols *μ_j_*(*r*, *θ*, *t*) and *n_j_*(*r*, *θ*, *t*) indicate, respectively, the electrochemical potential and the concentration (the number density) of the *j*-th ionic species, *μ_j_*_0_ is a constant term in *μ_j_*(*r*, *θ*, *t*), *e* is the elementary electric charge, *k* is Boltzmann’s constant, *T* is the absolute temperature, *ε*_r_ is the relative permittivity of the liquid, and *ε*_0_ is the permittivity of a vacuum. Equation (12) is the Poisson equation connecting the electric potential *ψ*(*r*, *θ*, *t*) outside the drop and the charge density *ρ*_e_(*r*, *θ*, *t*). For the drop interior, the electric potential *ψ*_I_(*r*, *θ*, *t*) satisfies the Laplace equation (Equation (14)). All these quantities-*p*(*r*, *θ*, *t*), *p*_I_(*r*, *θ*, *t*), *ψ*(*r*, *θ*, *t*), *ρ*_e_(*r*, *θ*, *t*), *μ_j_*(*r*, *θ*, *t*), and *n_j_*(*r*, *θ*, *t*) -depend on the spatial coordinates (*r*, *θ*) and time *t*.

Equations (5)–(8) correspond to the Navier–Stokes and continuity equations for an incompressible fluid under assumption (i). The term containing the drop velocity ***U**e^−iωt^* in Equations (5) and (6) appear because the coordinate system is defined in a frame of reference fixed to the moving drop. Equation (9) describes the velocity ***v**_j_*(*r*, *θ*, *t*) of the *j*-th ionic species, which is governed by both the bulk fluid motion ***u***(*r*, *θ*, *t*) and the spatial variation in the electrochemical potential *μ_j_*(*r*, *θ*, *t*), as given by Equation (10). Equation (11) represents the continuity equation for each ionic species.

Ions are adsorbed onto the drop surface. For the *j*-th adsorbed ionic species, let $n_{i}^{s}\left(\theta ,t\right)$, $\mu_{i}^{s}\left(\theta ,t\right)$, $v_{i}^{s}\left(\theta ,t\right)$, and $\lambda_{i}^{s}$ represent its surface number density, electrochemical potential, velocity, and drag coefficient, respectively. These surface quantities depend solely on *θ* and *t*, and obey the following relations:

(15) $$v_{i}^{s}\left(\theta ,t\right)=u\left(a,\theta ,t\right)-\frac{1}{\lambda_{i}^{s}}\nabla \mu_{i}^{s}\left(\theta ,t\right)$$

(16) $$\mu_{j}^{s}\left(\theta ,t\right)=\mu_{j0}^{s}+z_{j}e\psi \left(a,\theta ,t\right)+kTln\left[n_{j}^{s}\left(\theta ,t\right)\right]$$

(17) $$\nabla_{s}\cdot \left(n_{j}^{s}\left(\theta ,t\right)v_{j}^{s}\left(\theta ,t\right)\right)+n_{j}\left(a,\theta ,t\right)v_{jr}\left(a,\theta ,t\right)=0$$

(18) $$n_{j}^{s}\left(\theta ,t\right)=K_{j}n_{j}\left(a,\theta ,t\right)$$

(19) $$\sigma \left(\theta ,t\right)=\sum_{j=1}^{N}{z_{j}en}_{j}^{s}\left(\theta ,t\right)=\sum_{j=1}^{N}{K_{j}z_{j}en}_{j}\left(a,\theta ,t\right)$$

(20) $$\gamma \left(\theta ,t\right)=\gamma_{0}-kT\sum_{j=1}^{N}n_{j}^{s}\left(\theta ,t\right)=\gamma_{0}-kT\sum_{j=1}^{N}{K_{j}n}_{j}\left(a,\theta ,t\right)$$

Equation (15) describes that the surface flow $v_{i}^{s}\left(\theta ,t\right)$ of *j*-th ionic species adsorbed on the drop arises from two factors: the tangential liquid motion ***u***(*a*, *θ*, *t*) and the gradient of its electrochemical potential $\mu_{j}^{s}\left(\theta ,t\right)$, as defined in Equation (16). Equation (17) represents the continuity relation for the *j*-th adsorbed species on the drop interface, where ∇_s_ denotes the surface divergence operator. According to Equation (18), the *j*-th ion can be linearly adsorbed onto the drop surface with an adsorption coefficient *K_j_* (assumption (v)), leading to a surface charge density *σ*(*θ*, *t*) described by Equation (19). The coefficient *K_j_* is nonzero only for ions that can adsorb onto the drop surface, whereas it vanishes for non-adsorbing ions, such as those present in the background electrolyte. According to Equation (20), the surface tension of the drop comprises two parts: $\gamma_{0}$, representing the tension in the absence of adsorption, and an additional term accounting for the reduction in tension due to linear adsorption of ions.

### 2.2. Boundary Conditions

The liquid velocity ***u***(*r*, *θ*) is subject to the following boundary conditions at the drop surface (r=a) and in the region far from the drop (*r* → ∞). At the surface (r=a), the velocities inside and outside the drop, ***u***(*r*, *θ*) and ***u***_I_(*r*, *θ*), are continuous, and their normal components vanish, i.e.,

(21) $$u_{r}\left(a,\theta \right)=u_{Ir}\left(a,\theta \right)=0$$

(22) $$u_{\theta }\left(a,\theta \right)=u_{I\theta }\left(a,\theta \right)$$

Far from the drop, the velocity ***u***(*r*, *θ*) approaches −***U*** because the reference frame is attached to the drop, that is,

(23) $$u\left(r,\theta \right)\to -U\;as\;r\to \;\infty$$

The electric potentials *ψ*(*r*, *θ*, *t*) and *ψ*_I_(*r*, *θ*, *t*), and the ionic concentration *n_j_*(*r*, *θ*, *t*) of the *j*-th ionic species must satisfy the following boundary conditions:

(24) $$\psi_{I}\left(a,\theta ,t\right)=\psi \left(a,\theta ,t\right)$$

(25) $${\left.\varepsilon_{d}\frac{\partial \psi_{I}\left(r,\theta ,t\right)}{\partial r}\right|}_{r=a^{-}}-{\left.\varepsilon_{r}\frac{\partial \psi \left(r,\theta ,t\right)}{\partial r}\right|}_{r=a^{+}}=\frac{\sigma (\theta ,t)}{\varepsilon_{0}}=\frac{1}{\varepsilon_{0}}\sum_{j=1}^{N}{K_{j}z_{j}en}_{j}\left(a,\theta ,t\right)$$

where Equation (19) has been used. Equations (24) and (25) indicate, respectively, that the electric potential must remain continuous at the drop surface (*r* = *a*), and that a jump in its normal derivative across the drop surface corresponds to the surface charge density *σ*(*θ*, *t*) defined in Equation (19). Far from the drop, beyond the electrical double layer, the perturbations in the electric potential *ψ*(*r*, *θ*, *t*) and the concentration *n_j(_r*, *θ*, *t*) of the *j*-th ionic species due to the presence of the drop become negligible, so that we have

(26) $$\psi \left(r,\theta ,t\right)\to -Ee^{-i\omega t}rcos\theta \;\;as\;r\;\to \infty$$

(27) $$n_{i}\left(r,\theta ,t\right)\to n_{i}^{\infty }\;\;as\;r\;\to \infty$$

The tangential component of the stress tensor, which accounts for both hydrodynamic and Maxwell contributions as well as the Marangoni effect, must satisfy the continuity condition,

(28) $$\left\{\sigma_{r\theta }^{H}\left(a,\theta ,t\right)+\sigma_{r\theta }^{M}\left(a,\theta ,t\right)\right\}-\left\{\sigma_{Ir\theta }^{H}\left(a,\theta ,t\right)+\sigma_{Ir\theta }^{M}\left(a,\theta ,t\right)\right\}+\frac{1}{a}\frac{\partial \gamma (\theta ,t)}{\partial \theta }=0$$

where $\sigma_{r\theta }^{H}(r,\theta ,t)$ and $\sigma_{r\theta }^{M}(r,\theta ,t)$ represent the tangential components of the hydrodynamic and Maxwell stresses outside the drop, respectively, while $\sigma_{Ir\theta }^{H}(r,\theta ,t)$ and $\sigma_{Ir\theta }^{M}(r,\theta ,t)$ represent the corresponding stress components inside the drop.

The boundary condition for ***u*** far from the drop takes the same form as that for a spherical rigid particle obtained by Mangelsdorf and White [28], viz.,

(29) $$u(r,\theta )\to -U+\frac{a^{3}\left(\rho_{d}-\rho_{0}\right)}{3\rho_{0}r^{3}}\left[U-3\left(U\cdot \hat{r}\right)\hat{r}\right]\;\;as\;r\;\to \infty$$

where $\hat{r}=r/\left|r\right|$. The far-field boundary condition, given by Equation (23), must be replaced by Equation (29).

### 2.3. Equilibrium Distributions of Ionic Concentration, Charge, and Electric Potential, and Surface Charge Density in the Absence of an Applied Electric Field

We denote equilibrium quantities by the superscript (0), which depend *r* only, and assume that the ionic species obey the Boltzmann distribution. The equilibrium ionic concentration *n_j_*^(0)^(*r*) and space charge density *ρ*_e_^(0)^(*r*) at position ***r*** outside the drop are thus given by

(30) $$n_{j}^{\left(0\right)}\left(r\right)=n_{i}^{\infty }exp\left(-\frac{z_{j}e\psi^{\left(0\right)}\left(r\right)}{kT}\right)$$

(31) $$\rho_{e}^{\left(0\right)}\left(r\right)=\sum_{j=1}^{N}z_{j}en_{j}^{\left(0\right)}\left(r\right)$$

Here *ψ*^(0)^(*r*) represents the equilibrium electric potential and satisfies the following Poisson equation:

(32) $$∆\psi^{\left(0\right)}\left(r\right)=-\frac{\rho_{e}^{\left(0\right)}\left(r\right)}{\varepsilon_{r}\varepsilon_{0}}$$

Combining Equations (30)–(32), we obtain the Poisson-Boltzmann equations for *ψ*^(0)^(*r*), viz.,

(33) $$∆\psi^{\left(0\right)}\left(r\right)=-\frac{1}{\varepsilon_{r}\varepsilon_{0}}\sum_{j=1}^{N}z_{i}en_{i}^{\infty }exp\left(-\frac{z_{j}e\psi^{\left(0\right)}\left(r\right)}{kT}\right)$$

The boundary conditions for Equation (33) are

(34) $$\psi^{\left(0\right)}\left(a\right)=\zeta$$

(35) $$\psi^{\left(0\right)}\left(r\right)\to 0\;\;as\;\;r\to \infty$$

The equilibrium concentration (number density) of the *j*-th ionic species adsorbed onto the drop surface is represented by $n_{i}^{s,(0)}$ and is given by

(36) $$n_{j}^{s,(0)}=K_{j}n_{j}^{\left(0\right)}\left(a\right)=K_{j}n_{j}^{\infty }exp\left(-\frac{z_{j}e\zeta }{kT}\right)$$

The equilibrium surface charge density *σ*^(0)^ is thus given by

(37) $$\sigma^{\left(0\right)}=\sum_{j=1}^{N}{z_{j}en}_{j}^{s,(0)}=\sum_{j=1}^{N}K_{j}z_{j}en_{j}^{\left(0\right)}\left(a\right)=\sum_{j=1}^{N}K_{j}z_{j}en_{j}^{\infty }exp\left(-\frac{z_{j}e\zeta }{kT}\right)$$

In this situation, both $\psi_{I}^{\left(0\right)}$ and $n_{j}^{s,\left(0\right)}$ take constant values. This is because, when no electrolyte ions are present inside the droplet and the adsorbed ions are spread evenly over its surface in the absence of an external electric field ***E***, the electric potential inside the drop-determined from the Laplace equation (Equation (14))-becomes uniform throughout the interior. We thus obtain

(38) $${\left.\frac{d\psi^{\left(0\right)}(r)}{dr}\right|}_{r=a^{+}}=-\frac{\sigma^{\left(0\right)}}{\varepsilon_{r}\varepsilon_{0}}=-\frac{1}{\varepsilon_{r}\varepsilon_{0}}\sum_{j=1}^{N}K_{j}z_{j}en_{j}^{\infty }exp\left(-\frac{z_{j}e\zeta }{kT}\right)$$

For the low potential case, Equation (33) can be linearized to give

(39) $$∆\psi^{\left(0\right)}\left(r\right)=\kappa^{2}\psi^{\left(0\right)}\left(r\right)$$

where

(40) $$\kappa ={\left(\frac{\sum_{j=1}^{N}z_{j}^{2}e^{2}n_{j}^{\infty }}{\varepsilon_{r}\varepsilon_{0}kT}\right)}^{1/2}$$

is the Debye-Hückel parameter (1/*κ* is the Debye length), and Equation (38) reduces to

(41) $${\left.\frac{d\psi^{\left(0\right)}(r)}{dr}\right|}_{r=a^{+}}=-\frac{\sigma^{\left(0\right)}}{\varepsilon_{r}\varepsilon_{0}}=-\frac{1}{\varepsilon_{r}\varepsilon_{0}}\sum_{j=1}^{N}K_{j}z_{j}en_{j}^{\infty }$$

The solution to Equation (39) subject to Equations (34) and (35) is given by

(42) $$\psi^{\left(0\right)}\left(r\right)=\zeta \frac{a}{r}e^{-\kappa (r-a)}$$

with

(43) $$\zeta =\frac{a\sigma^{\left(0\right)}}{\varepsilon_{r}\varepsilon_{0}(1+\kappa a)}=\frac{a}{\varepsilon_{r}\varepsilon_{0}(1+\kappa a)}\sum_{j=1}^{N}K_{j}z_{j}en_{j}^{\infty }$$

and

(44) $$\sigma^{\left(0\right)}=\sum_{j=1}^{N}K_{j}z_{j}en_{j}^{\infty }$$

Equation (42) represents the potential distribution *ψ*^(0)^(*r*) around a weakly charged oil drop and Equations (43) and (44) are its zeta potential *ζ* and equilibrium surface charged density *σ*^(0)^, respectively.

### 2.4. Weak Field Approximation: Linearization

When the applied electric field ***E*** is weak, the deviations of *n_j_*(*r*, *θ*, *t*), $n_{j}^{s}\left(\theta \right)$, *ψ*(*r*, *θ*), *ψ*_I_(*r*, *θ*), *ρ*_e_(*r*, *θ*), *μ_j_*(*r*, *θ*), and $\mu_{j}^{s}\left(\theta \right)$ from their equilibrium values (i.e., those in the absence of the applied electric field ***E***) due to the applied field ***E*** are small. In other words, under a weak applied field, only small changes in the ion concentrations and electric potentials occur, which allows the governing equations to be linearized. In this case, we may write

(45) $$n_{j}\left(r,\theta ,t\right)=n_{i}^{\left(0\right)}\left(r\right)+\delta n_{i}\left(r\right)e^{-i\omega t}$$

(46) $${n_{i}^{s}\left(\theta ,t\right)=n}_{i}^{s,\left(0\right)}+\delta n_{i}^{s}\left(\theta \right)e^{-i\omega t}$$

(47) $$\psi \left(r,\theta ,t\right)=\psi^{\left(0\right)}\left(r\right)+\delta \psi \left(r,\theta \right)e^{-i\omega t}$$

(48) $$\psi_{I}\left(r,\theta ,t\right)=\psi_{I}^{\left(0\right)}+\delta \psi_{I}\left(r,\theta \right)e^{-i\omega t}$$

(49) $$\rho_{e}\left(r,\theta ,t\right)=\rho_{e}^{\left(0\right)}\left(r\right)+\delta \rho \left(r,\theta \right)e^{-i\omega t}$$

(50) $$\mu_{i}\left(r,\theta ,t\right)=\mu_{i}^{\left(0\right)}+\delta \mu_{i}\left(r,\theta \right)e^{-i\omega t}$$

(51) $$\mu_{i}^{s}\left(\theta ,t\right)=\mu_{i}^{s,\left(0\right)}+\delta \mu_{i}^{s}\left(\theta \right)e^{-i\omega t}$$

Here quantitates with superscript (0) represent those at equilibrium. By substituting Equations (45)–(51) into Equations (10), (12), (13), (18), and (25), we find the following relations between the small quantities:

(52) $${\delta \mu }_{j}\left(r,\theta \right)=z_{j}e\delta \psi \left(r,\theta \right)+\frac{kT\delta n_{j}\left(r,\theta \right)}{n_{j}^{\left(0\right)}\left(r\right)}$$

(53) $${\delta \rho }_{e}\left(r,\theta \right)=\sum_{j=1}^{N}z_{j}e{\delta n}_{j}\left(r,\theta \right)$$

(54) $$∆\delta \psi \left(r,\theta \right)=-\frac{\delta \rho_{e}\left(r,\theta \right)}{\varepsilon_{r}\varepsilon_{0}}$$

(55) $$\delta n_{j}^{s}\left(\theta \right)=K_{j}\delta n_{j}\left(a,\theta \right)$$

(56) $$\varepsilon_{d}{\left.\frac{\partial {\delta \psi }_{I}\left(r,\theta \right)}{\partial r}\right|}_{r=a^{-}}-\varepsilon_{r}{\left.\frac{\partial \delta \psi \left(r,\theta \right)}{\partial r}\right|}_{r=a^{+}}=\frac{\delta \sigma \left(\theta \right)}{\varepsilon_{0}}$$

From Equations (16), (46), (47), and (51), we obtain

(57) $$\delta \mu_{j}^{s}\left(\theta \right)=z_{j}e\delta \psi \left(a,\theta \right)+\frac{kTK_{j}\delta n_{j}\left(a,\theta \right)}{K_{j}n_{j}^{\left(0\right)}\left(a\right)}=z_{j}e\delta \psi \left(a,\theta \right)+\frac{kT\delta n_{j}\left(a,\theta \right)}{n_{j}^{\left(0\right)}\left(a\right)}$$

By comparing Equations (52) and (57), we obtain

(58) $$\delta \mu_{i}^{s}\left(\theta \right)={\delta \mu }_{i}\left(a,\theta \right)$$

Equation (28), which represents the tangential stress balance on the drop surface, thus reduces to

(59) $$\eta {\left.\left(\frac{1}{r}\frac{\partial u_{r}}{\partial \theta }+\frac{\partial u_{\theta }}{\partial r}-\frac{u_{\theta }}{r}\right)\right|}_{r=a^{+}}-\eta_{d}{\left.\left(\frac{1}{r}\frac{\partial u_{Ir}}{\partial \theta }+\frac{\partial u_{I\theta }}{\partial r}-\frac{u_{I\theta }}{r}\right)\right|}_{r=a^{-}}-\sum_{i=1}^{N}{K_{i}n^{\infty }e^{-z_{i}y\left(r\right)}\frac{1}{a}\frac{\partial }{\partial \theta }\delta \mu }_{i}\left(a,\theta \right)=0$$

It must be noted that Equation (59) does not depend on *δn_j_* or *δψ* separately, but on their combination, *δφ_j_*.

Similarly, the surface continuity conditions (Equation (7)) becomes

(60) $$\frac{1}{asin\theta }\frac{\partial }{\partial \theta }\left[sin\theta \left\{u_{\theta }\left(a,\theta \right)-\frac{1}{\lambda_{i}^{s}a}\frac{\partial }{\partial \theta }\delta \mu_{i}(a,\theta )\right\}\right]-{\left.\frac{1}{\lambda_{i}}\frac{\partial }{\partial r}\delta \mu_{i}(r,\theta )\right|}_{r=a^{+}}=0$$

Since *ψ*(*r*) → −*Er*cos*θ*, *ψ*^(0)^(*r*) → 0, *δψ*(*r*) → −*Er*cos*θ*, $n_{j}\left(r,\theta \right)\to n_{j}^{\infty }$, and *δn_j_*(r) → 0 as *r* → ∞, we obtain from Equation (52),

(61) $${\delta \mu }_{i}\left(r,\theta \right)\to -z_{i}eErcos\theta \;\;as\;r\;\to \infty$$

In Equations (45)–(51), the quantities marked with *δ*, together with ***u***(*r*, *θ*), ***u***_I_(*r*, *θ*), ***v**_j_*(*r*, *θ*), and $v_{j}^{s}\left(\theta \right)$ are all of order ***E***-that is, they vary linearly with the applied field ***E***. Hence, any products of such small quantities, which would contribute only at higher order in ***E***, are omitted. Taking the curl of Equation (25) to remove the pressure contribution, inserting Equations (55) and (60), and discarding higher-order small terms, we are led to the following result:

(62) $$\eta \nabla \times \nabla \times \nabla \times u\left(r\right)-i\omega \rho_{0}\nabla \times u\left(r\right)=\sum_{j=1}^{N}\nabla \delta \mu_{j}(r)\times \nabla n_{j}^{\left(0\right)}\left(r\right)$$

and by taking the curl of Equations (6) to eliminate the pressure terms ∇*p*_I_(r, *θ*), we obtain

(63) $$\eta_{d}\nabla \times \nabla \times \nabla \times u_{I}\left(r\right)-i\omega \rho_{0}\nabla \times u_{I}\left(r\right)=0$$

Similarly, from Equation (11), we obtain

(64) $$-i\omega \delta n_{j}\left(r\right)+\nabla \cdot \left(n_{j}^{\left(0\right)}\left(r\right)u\left(r\right)-\frac{1}{\lambda_{j}}n_{j}^{\left(0\right)}\left(r\right)\nabla \delta \mu_{j}\left(r\right)\right)=0\;$$

Further, symmetry considerations permit us to write

(65) $$u\left(r,\theta \right)=\left(-\frac{2}{r}h\left(r\right)Ecos\theta ,\;\frac{1}{r}\frac{d}{dr}(rh\left(r\right))Esin\theta ,\;0\right)\;$$

(66) $$u_{I}\left(r,\theta \right)=\left(-\frac{2}{r}h_{I}\left(r\right)Ecos\theta ,\;\frac{1}{r}\frac{d}{dr}(rh_{I}\left(r\right))Esin\theta ,\;0\right)$$

(67) $$\delta \mu_{j}\left(r,\theta \right)=-z_{j}e\phi_{j}\left(r\right)Ecos\theta$$

(68) $$\delta \psi \left(r\right)=-Y\left(r\right)Ecos\theta$$

where *h*(*r*), *h*_I_(*r*), *φ_j_*(*r*), and *Y*(*r*) depend on r only. Equations (65) and (66) automatically satisfy Equations (7) and (8), respectively. By substituting Equations (65)–(68) into Equations (62)–(64), we obtain the following linear equations for *h*(*r*), *φ_j_*(*r*), and *Y*(*r*):

(69) $$L\left[Lh\left(r\right)+\gamma^{2}h(r)\right]=G\left(r\right)\;$$

(70) $$L\left[Lh_{I}\left(r\right)+\gamma_{d}^{2}h_{I}(r)\right]=0$$

(71) $$L\phi_{j}(r)-\kappa^{2}\gamma_{j}\left\{\phi_{j}(r)-Y(r)\right\}=\frac{dy(r)}{dr}\left\{z_{j}\frac{d\phi_{j}(r)}{dr}-\frac{2\lambda_{j}}{e}\frac{h(r)}{r}\right\}$$

(72) $$LY(r)=\frac{1}{\varepsilon_{r}\varepsilon_{o}kT}\sum_{j=1}^{N}z_{j}^{2}e^{2}n_{j}^{\left(0\right)}\left(r\right)\left\{Y\left(r\right)-\phi_{j}(r)\right\}\;$$

with

(73) $$\gamma =\sqrt{\frac{i\omega \rho_{0}}{\eta }}=\left(i+1\right)\sqrt{\frac{\omega \rho_{0}}{2\eta }}=(i+1)\frac{1}{\delta }$$

(74) $$\gamma_{d}=\sqrt{\frac{i\omega \rho_{0}}{\eta }}=\left(i+1\right)\sqrt{\frac{\omega \rho_{d}}{2\eta_{d}}}=(i+1)\frac{1}{\delta_{d}}$$

(75) $$\gamma_{j}=-\frac{i\omega \lambda_{j}}{\kappa^{2}kT}\;\;$$

(76) $$L=\frac{d}{dr}\frac{1}{r^{2}}\frac{d}{dr}r^{2}=\frac{d^{2}}{dr^{2}}+\frac{2}{r}\frac{d}{dr}-\frac{2}{r^{2}}$$

(77) $$G\left(r\right)=-\frac{e}{\eta r}\frac{dy(r)}{dr}\sum_{j=1}^{N}z_{j}^{2}n_{j}^{\infty }e^{-z_{i}y\left(r\right)}\phi_{i}(r)$$

(78) $$g_{j}\left(r\right)=\frac{dy(r)}{dr}\left\{z_{j}\frac{d\phi_{j}(r)}{dr}-\frac{2\lambda_{j}}{e}\frac{h(r)}{r}\right\}$$

(79) $$y(r)=\frac{e\psi^{\left(0\right)}(r)}{kT}$$

where *δ* and *δ*_d_ are the penetration lengths in the liquid outside and inside the drop, respectively, L defined by Equation (76) is a linear differential operator and *y*(*r*) (Equation (79)) is the scaled equilibrium electric potential outside the drop.

The boundary conditions (21), (22), (59), and (29) for ***u***(*r*, *θ*) and ***u***_I_(*r*, *θ*) become

(80) $$h(a^{+})=0$$

(81) $$h_{I}(a^{-})=0$$

(82) $${\left.\frac{dh_{I}}{dr}\right|}_{r=a^{-}}={\left.\frac{dh}{dr}\right|}_{r=a^{+}}$$

(83) $$\eta_{d}{\left.\frac{d^{2}h_{I}}{dr^{2}}\right|}_{r=a^{-}}-\eta {\left.\frac{d^{2}h}{dr^{2}}\right|}_{r=a^{+}}=-\frac{1}{a}\sum_{j=1}^{N}{K_{j}z}_{j}en_{j}^{\infty }exp\left(-\frac{z_{i}e\zeta }{kT}\right)\phi_{i}(a)$$

(84) $$h\left(r\right)\to \frac{\mu (\omega )}{2}r+\frac{a^{3}\left(\rho_{d}-\rho_{0}\right)}{3\rho_{0}r^{2}}\mu (\omega )\;\;as\;r\;\to \infty$$

A solution of Equation (70) which does not have a singularity at r = 0 and satisfies Equation (82) is

(85) $$h_{I}\left(r\right)=A\left[r-\left\{\frac{\gamma_{d}rcos\left(\gamma_{d}r\right)-sin(\gamma_{d}r)}{\gamma_{d}acos\left(\gamma_{d}a\right)-sin(\gamma_{d}a)}\right\}\frac{a^{3}}{r^{2}}\right]$$

with *A* being a constant. By using Equation (85), we can combine Equations (82) and (83) to give the boundary condition involving *h*(*r*) and *φj*(*a*) only,

(86) $${\left.\frac{dh}{dr}\right|}_{r=a^{+}}-\frac{\eta Qa}{3\eta_{d}}{\left.\frac{d^{2}h}{dr^{2}}\right|}_{r=a^{+}}=-\frac{Q}{3\eta_{d}}\;\sum_{j=1}^{N}K_{j}z_{j}en_{j}^{\infty }exp\left(-\frac{z_{j}e\zeta }{kT}\right)\phi_{i}(a)$$

with

(87) $$Q=\frac{3\left[3\left(\gamma_{d}a\right)+\left\{{\left(\gamma_{d}a\right)}^{2}-3\right\}tan\left(\gamma_{d}a\right)\right]}{{\left(\gamma_{d}a\right)}^{3}-6\left(\gamma_{d}a\right)-3\left\{{\left(\gamma_{d}a\right)}^{2}-2\right\}tan\left(\gamma_{d}a\right)}$$

The boundary conditions for *δμ_j_*(*r*) can be written in terms of *φ_j_*(r) as follows.

(88) $${\left.\frac{d\phi_{j}(r)}{dr}\right|}_{r=a^{+}}+2K_{j}\left\{\frac{\lambda_{j}}{z_{j}ea}{\left.\frac{dh(r)}{dr}\right|}_{r=a^{+}}-\frac{\lambda_{j}}{\lambda_{j}^{s}}\frac{\phi_{i}(a)}{a^{2}}\right\}=0$$

(89) $$\phi_{j}(r)\to r\;\;as\;r\;\to \infty$$

Note here that Equation (88) corresponds to Equation (94) in our previous paper [55] for the static electrophoresis problem, which contained an error: $\lambda_{i}^{s}$ should be replaced by $\lambda_{i}^{s}a.$

### 2.5. General Electrophoretic Mobility Formula

The dynamic electrophoretic mobility *μ*(*ω*) = *U*/*E* of a spherical oil drop can be cal culated via

(90) $$\mu (\omega )=\frac{U}{E}=2\underset{r\to \infty }{\lim}\frac{h(r)}{r}$$

which is obtained from the solution to Equation (69). By solving Equation (69) subject to Equations (80), (84), and (86), and substituting the solution into Equation (90), the following general expression is derived for the dynamic electrophoretic mobility *μ*(*w*) of an ion-adsorbed spherical oil drop of radius *a* and zeta potential *ζ*:

(91) $$\mu \left(\omega \right)=\frac{2}{3\gamma^{2}\left\{L\left(a\right)-\Phi \right\}}\left[\int_{a}^{\infty }\left\{L\left(a\right)-L\left(r\right)\right\}G\left(r\right)dr-|\frac{\gamma^{2}Q(1-i\gamma a)}{{3\eta }_{d}+2\eta Q}|\;|\sum_{i=1}^{N}K_{i}z_{i}en_{i}^{\infty }exp\left(-\frac{z_{i}e\zeta }{kT}\right)\phi_{i}(a)\right]$$

with

(92) $$L\left(r\right)=H\left(r\right)-\frac{Q\eta }{3\eta_{d}+2Q\eta }\left(1-i\gamma a\right)\frac{\gamma^{2}r^{3}}{3a}$$

(93) $$L\left(a\right)=H\left(a\right)-\frac{Q\eta }{3\eta_{d}+2Q\eta }\left(1-i\gamma a\right)\frac{{\left(\gamma a\right)}^{2}}{3}$$

(94) $$H\left(r\right)=\left(1-i\gamma r\right)e^{i\gamma (r-a)}-\frac{\gamma^{2}r^{3}}{3a}$$

(95) $$H\left(a\right)=1-i\gamma a-\frac{\gamma^{2}a^{2}}{3}$$

(96) $$\Phi =\Gamma \left\{1+\frac{Q\eta }{3\eta_{d}+2Q\eta }\left(1-i\gamma a\right)\right\}$$

(97) $$\Gamma =\frac{2{\left(\gamma a\right)}^{2}\left(\rho_{0}-\rho_{d}\right)}{9\rho_{0}}$$

## 3. Results and Discussion

Equation (96) represents the main result of this work and gives a general expression for the dynamic electrophoretic mobility *μ*(*ω*) of an ion-adsorbed oil drop. This mobility expression is derived on the basis of the simplified Baygents and Saville theory [35]. The right-hand side of Equation (91) consists of two terms. The first term actually corresponds to the mobility of an ideally polarizable conducting drop such as a mercury drop. If both the Maxwell stress and the Marangoni stress contributions are absent in the tangential stress balance at the drop surface (Equation (28)), only the first term remains, and Equation (91) reduces to the mobility of a mercury drop. As in the static case [37], the magnitude of the mobility for such a drop is larger than that for a solid particle. For an oil drop with adsorbed ions, however, the Maxwell and Marangoni stresses contribute additionally, giving rise to the second term in the dynamic mobility. As a result, the magnitude of the mobility decreases. Consequently, for systems with the same zeta potential, the magnitude of the dynamic mobility follows the order: mercury drop > solid particle > oil drop.

Equation (91) is a general expression for the dynamic electrophoretic mobility *μ*(*ω*) of a spherical oil drop in an applied oscillating electric fields. In order to calculate the mobility value via Equation (91), one needs the function *G*(*r*), defined by Equation (77), which in turn requires the function *φ_j_*(*r*).

We consider two limiting cases. Consider first the case of *ω* → 0 (or *γ* → 0 and *γ_j_* → 0). In this case the applied electric field becomes static one so that the dynamic mobility *μ*(*ω*) tends to the usual static mobility *μ*(0). Indeed, in this limit Equation (91) tends to

(98) $$\mu \left(0\right)=\frac{a^{2}}{3}\int_{a}^{\infty }\left\{\frac{\eta_{d}}{3\eta_{d}+2\eta }-{\left(\frac{r}{a}\right)}^{2}+\frac{{2(\eta }_{d}+\eta )}{3\eta_{d}+2\eta }{\left(\frac{r}{a}\right)}^{3}\right\}G\left(r\right)dr-\frac{2}{3({3\eta }_{d}+2\eta )}\;\sum_{j=1}^{N}K_{j}z_{j}en_{j}^{\infty }exp\left(-\frac{z_{j}e\zeta }{kT}\right)\phi_{j}(a)$$

which is an expression for a static electrophoretic mobilit of a spherical oil drop with a radius *a* obtained in a previous paper (Equation (99) of Ref. [55]). Next consider the limiting case of *η*_d_ → ∞. In this limiting case, Equation (91) becomes

(99) $$\mu \left(\omega \right)=\frac{2}{3\gamma^{2}\left\{H\left(a\right)-\Gamma \right\}}\int_{a}^{\infty }\left\{H\left(a\right)-H\left(r\right)\right\}G\left(r\right)dr$$

which agrees with our previous result for the dynamic electrophoresis of a spherical rigid particle [30].

We next obtain an approximate analytical form of *μ*(*ω*) for a weakly charged oil drop. When the zeta potential is small, the equilibrium potential distribution is expressed by Equation (42). In addition, we assume that the drag coefficient $\lambda_{j}^{s}$ of ions adsorbed on the drop surface is much larger than that of the freely moving ions in the bulk, *λ_j_*. This assumption is generally valid for practical oil drops, because the adsorbed ions undergo substantial friction arising from their restricted motion along the interface and interactions with it, whereas ions in the surrounding electrolyte can move much more freely [35,53]. In other words, adsorbed ions move more slowly because their motion is constrained by the drop surface, which results in a larger effective drag than that experienced by bulk ions. We further assume that *λ_j_* is sufficiently small such that |*λ_j_*| « 1. Under these typical conditions, Equations (71) and (88) reduce to

(100) $$L\phi_{j}(r)=0$$

(101) $${\left.\frac{d\phi_{j}(r)}{dr}\right|}_{r=a^{+}}=0$$

from which, we obtain

(102) $$\phi_{i}\left(r\right)=r+\frac{a^{3}}{2r^{2}}$$

and the function *G*(*r*) (Equation (77)) becomes

(103) $$G\left(r\right)=-\frac{\varepsilon_{r}\varepsilon_{0}\kappa^{2}}{\eta }\left(1+\frac{a^{3}}{2r^{3}}\right)\frac{d\psi^{\left(0\right)}\left(r\right)}{dr}$$

By substituting Equation (103) together with the value of *φ_j_*(*a*) derived from Equation (102) into Equation (91), we obtain the following simplified expression for *μ*(*ω*), which is valid for low zeta potentials:

$$\mu \left(\omega \right)=\frac{\varepsilon_{r}\varepsilon_{0}\zeta \kappa^{2}}{\eta \gamma^{2}\left\{L\left(a\right)-\Phi \right\}}\left[e^{\left(\kappa -i\gamma \right)a}E_{5}\left(\left(\kappa -i\gamma \right)a\right)-i\gamma aE_{4}\left(\left(\kappa -i\gamma \right)a\right)-\frac{\gamma^{2}a^{2}}{3}E_{3}\left(\left(\kappa -i\gamma \right)a\right)\right.$$

(104) $$-L\left(a\right)e^{\kappa a}E_{5}\left(\kappa a\right)+\frac{2\gamma^{2}}{3\kappa^{2}}\left\{1-\frac{i\gamma \kappa a^{2}}{\left(\kappa -i\gamma \right)a}\right\}-\left.\frac{\eta \gamma^{2}Q(1-i\gamma a)(\kappa a+1)}{3\kappa^{2}(3\eta_{d}+2\eta Q)}\right]$$

where $E_{n}\left(\kappa a\right)={\left(\kappa a\right)}^{n-1}\int_{\kappa a}^{\infty }e^{-t}/t^{n}dt$ is the exponential integral of order *n*. The practical procedure for calculating the dynamic electrophoretic mobility *μ*(*ω*) is as follows. First, for a given oscillation frequency *ω* of the applied electric field, the mass density *ρ*_0_ of the surrounding liquid, and its viscosity *η*, the parameter *γ* is obtained from Equation (73). Similarly, using *ω* together with the mass density *ρ*_d_ and viscosity *η*_d_ of the drop, the parameter *γ*_d_ is calculated from Equation (74). With these quantities, along with the drop radius *a*, the Debye–Hückel parameter *κ*, the zeta potential *ζ*, and the relative permittivity *ε*_r_ of the medium, the dynamic mobility *μ*(*ω*) can be evaluated from Equation (104).

In the large *κa* limit (Smoluchowski limit), Equation (104) reduces to

(105) $$\mu (\omega )=\frac{\varepsilon_{r}\varepsilon_{o}\zeta }{\eta \left\{L\left(a\right)-\Phi \right\}}\left\{\frac{{3\eta }_{d}(1-i\gamma a)}{{3\eta }_{d}+2\eta Q}\right\}$$

while in the small *κa* limit (Hückel limit), Equation (104) reduces to

(106) $$\mu (\omega )=\frac{\varepsilon_{r}\varepsilon_{o}\zeta }{\eta \left\{L\left(a\right)-\Phi \right\}}\left\{\frac{{6\eta }_{d}+\left(3+i\gamma a\right)\eta Q}{3\left({3\eta }_{d}+2\eta Q\right)}\right\}$$

In the limit of *ω* → 0, Equation (104) tends to the static electrophoretic mobility of a liquid drop [55], viz.,

(107) $$\mu (0)=\frac{\varepsilon_{r}\varepsilon_{o}\zeta }{\eta }\left\{\left(\frac{{3\eta }_{d}}{{3\eta }_{d}+2\eta }\right)+2e^{\kappa a}E_{5}\left(\kappa a\right)-\left(\frac{15\eta_{d}}{{3\eta }_{d}+2\eta }\right)e^{\kappa a}E_{7}\left(\kappa a\right)\right\}$$

which further reduce the following Henry function for a spherical rigid particle in the limit of *ω* → 0 [6]:

(108) $$\mu (0)=\frac{\varepsilon_{r}\varepsilon_{o}\zeta }{\eta }\left\{1+2e^{\kappa a}E_{5}\left(\kappa a\right)-5e^{\kappa a}E_{7}\left(\kappa a\right)\right\}$$

Equation (104), which contains exponential integrals, is not always convenient for practical calculation of the dynamic electrophoretic mobility *μ*(*ω*) of an oil drop. To drive a simpler mobility formula, we start with the general dynamic electrophoretic mobility expression (Equation (91)), in which *G*(*r*) is replaced by its approximate form given in Equation (91). The first term on the right-hand side of Equation (91) then becomes

(109) $$-\frac{2\varepsilon_{r}\varepsilon_{0}\kappa^{2}}{3\eta \gamma^{2}\left\{L\left(a\right)-\Phi \right\}}\int_{a}^{\infty }\left\{L\left(a\right)-L\left(r\right)\right\}\left(1+\frac{a^{3}}{2r^{3}}\right)\frac{d\psi^{\left(0\right)}\left(r\right)}{dr}dr$$

We note that the term {*L*(*a*) − *L*(*r*)}*dψ*(*r*)^(0)^/*dr* in the integrand of Equation (91) exhibits a sharp maximum around *r* ≈ *a* + *δ*/*κ*, where *δ* is a constant of order unity, and becomes zero at the both integration limits *r* = *a* and *r* → ∞, and that the function (1 + *a*^3^/2*r*^3^) in Equation (109) varies slowly as compared with {*L*(*a*) − *L*(*r*)}*dψ*^(0)^/*dr*. This is due to the fact that the electrical double layer surrounding the drop is restricted to a thin region between *r* = *a* and *r* ≈ *a* + 1/*κ*. Therefore, in the term $\left(1+a^{3}/2r^{3}\right)$, r can be approximately replaced by *a* + *δ*/*κ*, allowing it to be taken outside the integral [37]. We found that setting *δ* = 2 gives the correct limiting forms for both κa→∞ and κa→0. Thus, the following simple approximate expression for the mobility has been derived:

$$\mu \left(\omega \right)=\frac{\varepsilon_{r}\varepsilon_{0}\zeta }{\eta \left\{L\left(a\right)-\Phi \right\}}\left[\frac{\left\{3\eta_{d}+\eta Q(\kappa a+1)\right\}\left\{\kappa -i\gamma (\kappa a+1)\right\}-\eta \gamma^{2}aQ(\kappa a+1)}{3(3\eta_{d}+2\eta Q)\left(\kappa -i\gamma \right)}\left\{1+\frac{1}{2(1+\frac{2}{\kappa a}{)}^{3}}\right\}\right.$$

(110) $$-\left.\frac{\eta \gamma^{2}Q(1-i\gamma a)(\kappa a+1)}{3\kappa^{2}(3\eta_{d}+2\eta Q)}\right]$$

Note that for the static electrophoretic mobility of a mercury drop, choosing *δ* = 1.86 yields a more accurate result, with errors of less than 1% [35].

**Figure 2:**
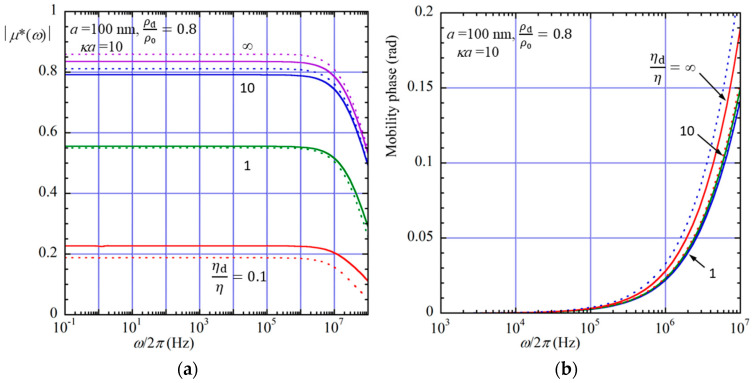
Magnitude (**a**) and phase (**b**) of the scaled dynamic electrophoretic mobility μ*(*ω*), defined as μ*(*ω*) = *μ*(*ω*)/(*ε*_r_*ε*_0_*ζ*/*η*), of a spherical liquid drop of radius *a*=100 nm in an aqueous electrolyte solution at 25 °C (*η* = 0.89 mPa · s and *ρ*_0_ = 0.997 × 10^3^ kg m^−3^) as functions of the frequency *ω*/2*π* (Hz) of the applied electric field. The solid lines represent the results calculated from Equation (104), and the dotted lines show the approximate results obtained from Equation (110) for several values of the viscosity ratio *η*_d_/*η* at the mass density ratio *ρ*_d_/*ρ*_0_ = 0.8 and *κa* = 10.

**Figure 3:**
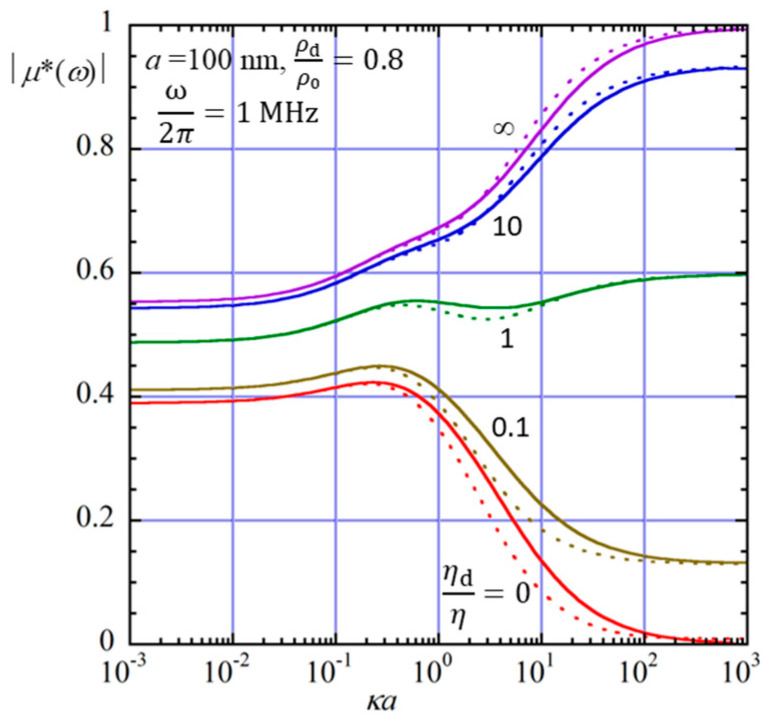
Magnitude of the scaled dynamic electrophoretic mobility μ*(*ω*), defined as μ*(*ω*) = *μ*(*ω*)/(*ε*_r_*ε*_0_*ζ*/*η*), of a spherical liquid drop of radius *a* = 100 nm in an aqueous electrolyte solution at 25 °C (*η* = 0.89 mPa · s and *ρ*_0_ = 0.997 × 10^3^ kg m^−3^) as a function of *κa*. The solid lines represent the results calculated from Equation (104), and the dotted lines show the approximate results obtained from Equation (110) for several values of the viscosity ratio *η*_d_/*η* at the mass density ratio *ρ*_d_/*ρ*_0_ = 0.8 and *ω*/2*π* = 1 MHz.

The results calculated by Equation (104) and its approximate form, Equation (110), are shown in Figure 2a,b and Figure 3.

Figure 2a,b illustrate the magnitude (a) and phase (b) of the scaled dynamic electrophoretic mobility μ*(*ω*), which is defined as μ*(*ω*) = *μ*(*ω*)/(*ε*_r_*ε*_0_*ζ*/*η*), of a spherical oil drop of radius *a* = 100 nm in an aqueous electrolyte solution at 25 °C (*η* = 0.89 mPa · s and *ρ*_0_ = 0.997 × 10^3^ kg m^−3^) for several values of *η*_d_/*η* at *ρ*_d_/*ρ*_0_ = 0.8 as a function of *ω*/2*π* at *κa* = 10.

Figure 3 shows μ*(*ω*) as a function of *κa* at *ω*/2*π* = 1 MHz. The solid lines represent the results calculated from Equation (104), and the dotted lines show the approximate results obtained from Equation (110). Figure 2a shows that the magnitude of *μ*(*ω*) remains almost constant up to approximately 1 MHz, taking a value close to the static electrophoretic mobility. When the frequency exceeds about 1 MHz, however, the magnitude of *μ*(*ω*) begins to decrease rapidly. In contrast, Figure 2b illustrates that the phase of *μ*(*ω*) is nearly zero up to around 0.1 MHz, indicating that the drop motion is in phase with the applied field. At higher frequencies, the phase increases sharply, showing a noticeable lag of the electrophoretic response relative to the applied electric field.

At low frequencies, both the ionic atmosphere and the fluid flow around the drop can follow the oscillation of the applied electric field. As a result, the mobility remains almost identical to the steady (static) electrophoretic mobility, and no phase shift is observed. When the frequency becomes comparable to the characteristic relaxation frequency of the electric double layer (on the order of 1 MHz in the present system), the redistribution of ions cannot keep up with the field oscillation. Consequently, the magnitude of *μ*(*w*) starts to decrease, and a phase lag develops. The rapid increase in the phase above about 0.1 MHz suggests that the electro-hydrodynamic coupling inside and outside the drop becomes increasingly out of phase with the driving field. The dependence on the viscosity ratio implies that the internal flow within the drop also plays a role in determining the characteristic frequency at which the mobility amplitude and phase start to vary. Drops with higher internal viscosity tend to exhibit a smaller amplitude and a delayed phase response, consistent with the reduced internal fluid motion.

Figure 3 illustrates how μ*(*ω*) depends on *κa* (the ratio of the drop radius *a* to the Debye length 1/*κ*). This function approaches the Henry function for a spherical rigid particle in the limit of *ω* → 0 and *η*_d_ → ∞. Thus, μ*(*ω*) as a function of *κa* can be regarded as the Henry function in the dynamic electrophoreses of an oil drop. These figures also show that Equation (110) provides a good approximation to Equation (104) with tolerable errors.

Finally, it should be noted that electroacoustic techniques such as electrokinetic sonic amplitude (ESA) and colloid vibration potential (CVP) measure signals that are directly influenced by the frequency-dependent electrophoretic mobility. The present analytical expression for the dynamic mobility therefore provides a basis for comparison with experimental data obtained for emulsions and other soft dispersed systems. Such comparisons would be helpful for further validation and application of the present theory.

## 4. Conclusions

In this study, a general theoretical expression for the dynamic electrophoretic mobility of a liquid drop in an electrolyte solution was derived on the basis of the simplified Baygents and Saville theory. The resulting equation (Equation (91)) consists of two terms: the first represents the mobility of a mercury drop, which can be treated as an ideally polarizable conducting drop, while the second arises from the contributions of the Maxwell and Marangoni stresses that become significant when ions are adsorbed at the oil–water interface. As a result, the magnitude of the mobility decreases with increasing influence of these interfacial stresses, leading to the relation: mercury > solid particle > ion-adsorbing oil drop for systems with the same zeta potential.

Based on the general mobility expression, we also derived an approximate expression valid for low-zeta-potential conditions (Equation (104)), which involves exponential integrals. Furthermore, a simpler expression for the mobility, free from exponential integrals and accurate within tolerable errors, was obtained (Equation (110)).

This study focuses on weakly charged, spherical oil drops. Future work may explore systems beyond this scope, such as non-spherical drops or drops with stronger interfacial ion adsorption.

## Data Availability

The original contributions presented in this study are included in the article. Further inquiries can be directed to the corresponding author.
